# The Fanconi anemia pathway and ubiquitin

**DOI:** 10.1186/1471-2091-8-S1-S10

**Published:** 2007-11-22

**Authors:** Céline Jacquemont, Toshiyasu Taniguchi

**Affiliations:** 1 Divisions of Human Biology and Public Health Sciences, Fred Hutchinson Cancer Research Center, 1100 Fairview Ave. N., C1-015, Seattle, WA 98109-1024, USA

## Abstract

Fanconi anemia (FA) is a rare genetic disorder characterized by aplastic anemia, cancer/leukemia susceptibility and cellular hypersensitivity to DNA crosslinking agents, such as cisplatin. To date, 12 FA gene products have been identified, which cooperate in a common DNA damage-activated signaling pathway regulating DNA repair (the FA pathway). Eight FA proteins form a nuclear complex harboring E3 ubiquitin ligase activity (the FA core complex) that, in response to DNA damage, mediates the monoubiquitylation of the FA protein FANCD2. Monoubiquitylated FANCD2 colocalizes in nuclear foci with proteins involved in DNA repair, including BRCA1, FANCD1/BRCA2, FANCN/PALB2 and RAD51. All these factors are required for cellular resistance to DNA crosslinking agents. The inactivation of the FA pathway has also been observed in a wide variety of human cancers and is implicated in the sensitivity of cancer cells to DNA crosslinking agents. Drugs that inhibit the FA pathway may be useful chemosensitizers in the treatment of cancer.

**Publication history: **Republished from Current BioData's Targeted Proteins database (TPdb; ).

## Protein pathway in the disease

### Fanconi anemia

Fanconi anemia (FA) is a rare autosomal or X-linked recessive disease characterized by chromosomal instability and cancer susceptibility. All FA complementation groups (discussed below) except FA-B are inherited autosomally [1,2]. FA prevalence is estimated to be 1–5 per million and the frequency of heterozygous carriers to be 1 in 300 [3,4]. Notwithstanding the small number of FA patients, this disease constitutes a dynamic research area because the FA pathway plays a crucial role in preventing genomic instability and provides an attractive model for understanding the interplay between DNA repair and ubiquitin biology in tumorigenesis and cancer therapy (reviewed in [5-15]).

The clinical course and the treatment of FA have been extensively reviewed elsewhere [3,4,16]. Clinically, FA is characterized by childhood onset aplastic anemia, increased cancer/leukemia susceptibility and developmental defects. Typically, FA patients develop bone marrow failure leading to aplastic anemia during the first decade of life and at least 20% develop malignancies. Most commonly, these include acute myelogenous leukemia and myelodysplastic syndrome, but also head and neck squamous cell carcinoma, gynecological squamous cell carcinoma, esophageal carcinoma, and liver, brain, skin and renal tumors [17-19]. FA subtypes FA-D1 and FA-N are associated with increased predisposition to medulloblastoma, Wilms' tumor and acute leukemia in early childhood, and are clinically different from the other FA subtypes [20-24]. Common developmental defects observed in FA are short stature, developmental disability and abnormalities of the skin, upper extremities, head, eyes, kidneys and ears [3]. In addition, male FA patients often present abnormal gonads [3].

FA cells are hypersensitive to treatment with DNA crosslinking agents such as cisplatin, mitomycin C (MMC), melphalan and diepoxybutane [25], leading to increased chromosome breakage [25,26].

### The Fanconi anemia pathway

#### Proteins involved in the Fanconi anemia pathway

**Figure 1:**
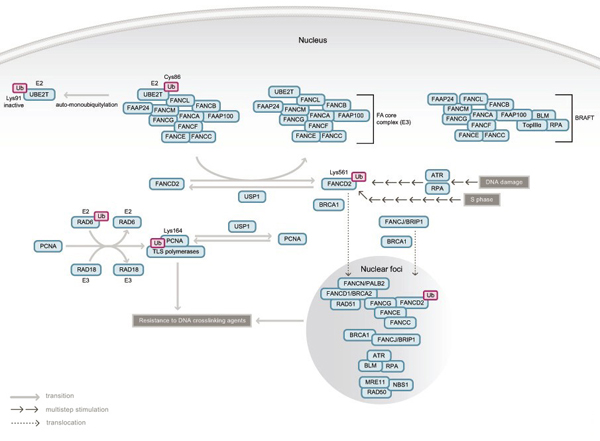
**Current model of the Fanconi anemia pathway**. (Adapted with modifications from “The molecular pathogenesis of Fanconi anemia: recent progress” Taniguchi T and D'Andrea AD. Blood, Jun 2006; 107: 4223 – 4233. [6]) Eight FA proteins (FANC-A, -B, -C, -E, -F, -G, -L and -M), a FANCM-interacting protein called FAAP24 and an unidentified factor (FAAP100) form a nuclear complex (the FA core complex) with E3 ubiquitin ligase activity. FANCL is the catalytic subunit of the complex and directly interacts with the E2 ubiquitin conjugating enzyme UBE2T through its PHD/RING domain. UBE2T can be inactivated by auto-monoubiquitylation on lysine 91 (K91), but is suggested to form a thioester intermediate with ubiquitin through it's catalytic cysteine 86 (C86) when taking part in the ubiquitylation of FANCD2 (see below). The FA core complex, BLM, RPA and topoisomerase IIIα form a larger super-complex called BRAFT. Note that for diagrammatic purposes the FA core complex is shown here interacting with the FAAP100 subunit, but in reality the quaternary structure of the BRAFT complex is unknown. In response to exogenous DNA damage, or during normal S phase progression, the FANCD2 protein is monoubiquitylated on lysine 561 (K561) in an FA core complex- and UBE2T-dependent manner. DNA damage-induced monoubiquitylation of FANCD2 also requires ATR and RPA. Monoubiquitylation of FANCD2 targets the protein into nuclear foci where it co-localizes with BRCA1, FANCD1/BRCA2, FANCN/PALB2, RAD51, FANCJ/BRIP1 and other proteins. At least some components of the FA core complex (FANCC, FANCE and FANCG) also form nuclear foci. All of these factors are required for cellular resistance to DNA crosslinking agents. Monoubiquitylation of PCNA on lysine 164 (K164) requires RAD6 as an E2 and RAD18 as an E3, but not the FA core complex. In turn, modification of PCNA causes recruitment of translesion synthesis (TLS) DNA polymerases at the site of stalled replication forks. USP1 deubiquitylates both PCNA and FANCD2, negatively regulating both the Fanconi anemia pathway and monoubiquitylation of PCNA.

FA can be divided into at least thirteen complementation groups (FA-A, -B, -C, -D1, -D2, -E, -F, -G, -I, -J, -L, -M and -N), of which the responsible genes for all groups except FA-I have been identified (summarized in Table 1) [6,23,24,27-30]. All FA proteins are required for cellular resistance to DNA crosslinking agents [6] and are considered to cooperate in a common pathway (the FA pathway) that regulates the sensing, signaling and/or repair of interstrand DNA crosslinks (Figure 1). FA proteins are closely related to the breast/ovarian cancer susceptibility genes products BRCA1 and BRCA2, and to their partner proteins, as described below. *FANCD1* (mutated in FA-D1) is identical to *BRCA2*[31] and the most recently identified FA gene, *FANCN,* is in fact *PALB2* (partner and localizer of BRCA2) [23,24], a crucial regulator of the BRCA2 protein [32]. Additionally, *FANCJ* is identical to *BACH1/BRIP1*[29,30,33], a DNA helicase that interacts directly with BRCA1. Furthermore, FANCD2, FANCD1/BRCA2, FANCN/PALB2 and BRCA1 colocalize in nuclear foci at the site of DNA damage [32][34,35] and BRCA1 itself is required for efficient nuclear foci formation of FANCD2 [34,36]. In light of these interplays between FA and BRCA proteins, the FA pathway is also called the “FA-BRCA pathway” [5] or “FA-BRCA network” [11].

**Table 1:** The responsible genes for Fanconi anemia

Subtype	Responsible gene	Protein (kDa)	Requirement for FANCD2 monoubiquitylation	Function of the protein, etc.	Homologs in						
					
					**Yeast**	**Plant**	**Fly**	**Worm**	**Fish**	**Amphibian**	**Human, mouse, chicken**
					*S. cerevisiae*	*A. thaliana*	*D. melanogaster*	*C. elegans*	*D. rerio*	*X. laevis*	
A	*FANCA*	163	+	FA core complex					+ [123]	+ [124]	+
B	*FANCB*	95	+	FA core complex					+ [123]	+ [124]	+
C	*FANCC*	63	+	FA core complex					+ [123]	+ [124]	+
D1	*FANCD1/BRCA2*	380	-	RAD51 recruitment				+ [135]	+ [123]	+ [124]	+
D2	*FANCD2*	155,162	+	monoubiquitylated protein		+ [133]	+ [27,125]	+ [126]	+ [123,127]	+ [124]	+
E	*FANCE*	60	+	FA core complex					+ [123]	+ [124]	+
F	*FANCF*	42	+	FA core complex					+ [123]	+ [136]	+
G	*FANCG/XRCC9*	68	+	FA core complex					+ [123]	+ [124]	+
I	not identified	?	+	?							
J	*FANCJ/BACH1/BRIP1*	130	-	5′→3′ DNA helicase/ATPase	+ [132]			+ [126]			+
L	*FANCL/PHF9/POG*	43	+	FA core complex, E3 ubiquitin ligase		+ [134]	+ [27,125]		+ [123]	+ [124]	+
M	*FANCM/Hef*	250	+	FA core complex, ATPase/translocase, DNA helicase motifs	+ [27]		+ [125]	+ [126]			+
N	*FANCN/PALB2*	130	-	regulation of BRCA2 localization					+	+	+

(Adapted with modifications from “The molecular pathogenesis of Fanconi anemia: recent progress” Taniguchi T and D'Andrea AD. Blood, Jun 2006; 107: 4223–4233. [6])

FANC protein homologs shown in the table are limited to the ones reported in the literature [27,123–127,132–136].

#### The FA core complex and monoubiquitylation of FANCD2

Ubiquitin plays a crucial role in the regulation of the FA pathway. Eight FA proteins (FANCA, FANCB, FANCC, FANCE, FANCF, FANCG, FANCL and FANCM), a newly identified FANCM-interacting protein called FAAP24 (FANCA-associated polypeptide) [137] and an unidentified factor called FAAP100 form a nuclear protein complex (the FA core complex) required for monoubiquitylation of FANCD2 on lysine 561 [27,34,37,38]. One of the components of the FA core complex, FANCL, has a PHD (plant homeodomain) finger/RING finger domain exhibiting auto-ubiquitin ligase activity *in vitro*[38][39-41]. FANCL associates through its PHD/RING finger domain with UBE2T, a ubiquitin conjugating enzyme (E2), which is also required for *in vivo* FANCD2 monoubiquitylation [42]. Taken together, the FA core complex is assumed to constitute a multi-subunit E3 ubiquitin ligase complex for FANCD2, in which FANCL is the catalytic E3 ubiquitin ligase subunit. The catalytic activity of this complex appears to be regulated through the inhibitory auto-monoubiquitylation of UBE2T, stimulated in the presence of FANCL [42]. However, as the direct monoubiquitylation of FANCD2 by the FA core complex has not been reconstituted so far *in vitro*, the existence of other E2 and E3 enzymes responsible for FANCD2 monoubiquitylation (and somehow controlled by the FA core complex, the E2 activity of UBE2T and the E3 activity of FANCL) cannot be ruled out formally.

Even though a direct DNA binding activity has been demonstrated for unmodified FANCD2 *in vitro*[43], monoubiquitylation of the protein is required for its translocation to chromatin *in vivo* and nuclear foci formation at the site of DNA damage, as well as for cellular resistance to DNA crosslinking agents [34,44]. FANCD2 monoubiquitylation and nuclear foci formation occur in response to DNA damaging agents (ionizing radiation, UV light irradiation, DNA crosslinking agents, hydroxyurea, etc.) and during S phase of the cell cycle even in the absence of exogenous DNA damage [34,45]. A DNA damage-activated signaling kinase, ATR, and a single-strand DNA binding protein complex, RPA, are required for DNA damage-inducible monoubiquitylation and foci formation of FANCD2, indicating an upstream role for these factors in the activation of the FA pathway [46]. The exact signal and activation cascade required for FANCD2 monoubiquitylation, however, remain elusive. The existence of a specific FANCD2 receptor responsible for recruitment of the monoubiquitylated protein to chromatin has also been proposed [44], but not demonstrated to date.

#### Roles for the FA core complex in DNA enzymatic processing

The FA core complex is not simply the ubiquitin ligase complex for FANCD2. In addition to its requirement for FANCD2 monoubiquitylation, the FA core complex is required for the translocation into chromatin of monoubiquitylated FANCD2, and for cellular resistance to interstrand DNA crosslinks even if FANCD2 is localized in chromatin, as elegantly demonstrated in chicken DT40 cells using FANCD2–monoubiquitin and FANCD2–histone H2B chimeric proteins [47]. In addition, one component of the FA core complex, FANCM, harbors DNA helicase motifs, a degenerate nuclease motif and *in vitro* DNA-stimulated ATPase and translocase activities [27]. The newly identified FAAP24 protein, in complex with FANCM, has been shown to preferentially bind to ssDNA and branched DNA structures [137]. Speculatively, FANCM DNA translocase activity could play an important role in displacing the FA core complex along the DNA, allowing DNA damage recognition, or FAAP24 specificity for ssDNA structures may target FANCM and the FA core complex to abnormal, branched DNA structures. In summary, the FA core complex itself can interact with DNA and displays some important functions outside of FANCD2 monoubiquitylation.

Furthermore, the FA core complex forms a larger complex with BLM, RPA and topoisomerase IIIα called BRAFT (for BLM, RPA, FA, and topoisomerase IIIα). Although the functional relevance of BRAFT is not clear, it harbors a DNA unwinding activity potentially relevant for DNA repair [37].

#### Negative regulation of the Fanconi anemia pathway by FANCD2 deubiquitylation

FANCD2 monoubiquitylation is a regulated and reversible process. USP1, a deubiquitylating enzyme, removes ubiquitin from monoubiquitylated FANCD2, therefore negatively regulating the FA pathway [48]. Interestingly, USP1 also deubiquitylates monoubiquitylated PCNA (proliferating cell nuclear antigen), a DNA polymerase processivity factor [49]. PCNA monoubiquitylation leads to the switch from a replicative polymerase to a translesion synthesis (TLS) polymerase at the site of stalled replication forks [50] and participates in the bypass of DNA lesion. Monoubiquitylation of PCNA is dependent on RAD6 (an E2) and RAD18 (an E3) [51], but is independent of the FA core complex. Therefore, these two pathways (the FA pathway and TLS activation through PCNA monoubiquitylation) have different activation mechanisms, but share a common shutoff mechanism.

#### The Fanconi anemia pathway and DNA repair

Interestingly, some TLS polymerases are also implicated in cellular resistance to interstrand DNA crosslinks [52]. For example, mutations in TLS polymerase-encoding *REV3* or *REV1* have been shown to be epistatic with *FANCC* mutations for crosslinker hypersensitivity in chicken DT40 cells [53]. Furthermore, REV1 and FANCD2 colocalize in nuclear foci [53], which could suggest a possible interaction between them. The possible interplay of FA proteins and TLS polymerases in a common pathway regulating the bypass of DNA damage, however, remains to be clarified.

One of the most important issues to clarify concerning the role of the FA pathway in DNA repair is how FA proteins (the FA core complex, FANCD2, FANCD1/BRCA2, FANCN/PALB2, FANCJ/BRIP1/BACH1) and other factors (RAD51, BRCA1, BLM, TLS polymerases etc.) cooperate to sense, signal and repair DNA crosslinks.

In nuclear foci, FANCD2 colocalizes with factors known to be involved in DNA repair and/or DNA damage response, such as BRCA1, FANCD1/BRCA2, FANCN /PALB2, RAD51, BLM, RPA and ATR [32,34-36,45,46]. FANCD2 also partially colocalizes with FANCJ/BRIP1/BACH1 [33] and NBS1, part of the MRE11–RAD50–NBS1 complex implicated in the recognition and early signalization of damaged DNA [54]. Among the subunits of the FA core complex, at least FANCC and FANCE are reported to colocalize with FANCD2 in nuclear foci [47,55], and FANCG with BRCA2 and RAD51 [56]. All these factors are required for cellular resistance to interstrand DNA crosslinking agents.

Among FA proteins, FANCD1/BRCA2 and FANCJ/BRIP1/BACH1 are neither part of the FA core complex nor required for FANCD2 monoubiquitylation or foci formation [28,31]. They may work downstream of FANCD2 monoubiquitylation and/or in another pathway. Interestingly, these proteins have been strongly linked to DNA double-strand breaks repair by homologous recombination (HR) [33,57]. Specifically, FANCD1/BRCA2 regulates HR by controlling the activity of RAD51, the eukaryotic homolog of bacterial RecA [57,58]. FANCD1/BRCA2 stability and localization in nuclear structures (chromatin and nuclear matrix) is in turn regulated by FANCN/PALB2 [32]. Whether FANCD1/BRCA2, FANCN/PALB2 and RAD51 act downstream or independently of monoubiquitylated FANCD2 and other FA proteins remains unclear. Evidence in favor of a regulatory role for the FA pathway on FANCD1/BRCA2 and RAD51 function comes from reports that monoubiquitylated FANCD2 is required for both the loading of FANCD1/BRCA2 onto damaged chromatin and the increase in FANCD1/BRCA2 and RAD51 nuclear foci in response to DNA damage [35]. However, the requirement for the FA core complex and FANCD2 in efficient RAD51 foci formation in response to DNA damage [35,59,60], as well as the efficiency of HR in FA cells (reviewed in reference [6]), remain controversial. FANCD1/BRCA2- and FANCN/PALB2-deficient cells have a clear defect in RAD51 foci formation and HR efficiency [23,32,57,61-63]. In other FA cells, at least mild defects in HR have been documented [33,53,62,64-67], although other reports show normal HR efficiency in these cells [68-70].

FANCJ/BRIP1/BACH1 has DNA-dependent ATPase activity and 5′–3′ DNA helicase activity, and directly binds to the phospho-protein binding BRCT (BRCA1 carboxyl-terminal domain [72,73]) of BRCA1 [71]. How FANCJ/BRIP1/BACH1 interacts with other FA proteins has not been reported, except for partial colocalization with FANCD2 in nuclear foci [33]. Though RAD51 foci formation is normal in FA-J cells (deficient in FANCJ/BRIP1/BACH1) [63], HR efficiency appears to be affected [33].

Taken together, these evidences suggest that FANCD2 plays a role in regulating DNA repair (especially of interstrand DNA crosslinks) in chromatin in cooperation with BRCA1, FANCD1/BRCA2, FANCJ/BRIP1/BACH1, the FA core complex and other factors, but the precise mechanism by which this occurs remains unknown. The highly regulated process of FANCD2 monoubiquitylation could play a crucial role in orienting the repair of DNA damage to HR and/or in attracting TLS polymerases to sites of DNA damage.

#### Other roles for the Fanconi anemia proteins outside of DNA repair

Several FA proteins function outside of DNA crosslink repair. For example, the phosphorylation of FANCD2 at serine 222 by ATM kinase in response to ionizing radiation is required for the establishment of IR-induced intra-S phase checkpoint, but is not required for resistance to DNA crosslinking agents [74]. Additionally, FANCC (localized in both the cytoplasm and nucleus) is implicated in several cytoplasmic functions, such as JAK/STAT and apoptotic signaling [75-80].

### Alterations in the Fanconi anemia pathway in human cancer in the general (non-FA) population

FA patients have an increased risk of developing leukemia and solid tumors, but alterations in the FA pathway have also been reported in a wide variety of human cancers in the general (non-FA) population [81-94] (reviewed in references [6,10]). Abnormalities in *BRCA1* and *BRCA2* in cancer have been reviewed elsewhere [95]. Methylation of *FANCF*, leading to its decreased expression, has been reported in ovarian cancer [81,82], breast cancer [84], non-small cell lung cancer [85], cervical cancer [86], testicular cancer [87], head and neck squamous cell carcinoma [85], and granulosa cell tumors of the ovary [83]. Inherited and somatic mutations of *FANCC* and *FANCG* have been detected in a subset of young onset pancreatic cancer [89,96]. Additionally, inherited mono-allelic mutations in *BACH1/BRIP1/FANCJ* and *FANCN/PALB2* have been recently implicated in breast cancer predisposition (familial breast cancer) [97][23,24], suggesting that these genes may be the low penetrance breast/ovarian cancer susceptibility genes researchers have been looking for to explain the non-BRCA1/non-BRCA2 cases. Because the integrity of the FA pathway is crucial for cellular resistance to interstrand DNA crosslinking agents (cisplatin, MMC, melphalan, etc.), tumors with defects in the pathway may be hypersensitive to these agents. In fact, in some cancer cell lines (human ovarian cancer cell lines (TOV-21G and 2008) [81], human myeloma cell lines (8226 and U266) [98] and a human pancreatic cancer cell line (PL11) [99]), the integrity of the FA pathway is a determinant of cisplatin (or melphalan) resistance *in vitro* or *in vivo* (mouse xenograft model) [81,98-100].

## Disease models, knockouts, assays

Generation of various knockout murine models for FA (*Fanca*[101,102]*, Fancc*[103,104], *Fancg*[105,106], *Fancd2*[107], *Fanca*-*Fancc* double [108], *Fancl*/*Pog*[109] and *Fancd1/Brca2 *[110] ) have been reported. These FA mice display similar phenotypes to that of human FA patients, for example chromosome instability, defective germ cell development and sensitivity to DNA crosslinking agents. Although FA mice do not spontaneously develop bone marrow failure (except for mouse models of the FA-D1 group, harboring homozygous hypomorphic mutations in *Fancd1/Brca2*[110]), treatment with MMC causes bone marrow failure in *Fancc* knockout mice [111]. The reason for the absence of spontaneous bone marrow failure in FA mice is still unknown. Several FA mice (*Fancd2*[107]*, Fanca*[102] and *Fancc *[112]) are reported to develop tumors including adenocarcinoma, lymphoma, sarcoma and ovarian granulosa cell tumor. In addition, many *Brca1-* and *Brca2-*deficient mouse models with cancer susceptibility have been generated (reviewed in reference [113]). FA mice are useful tools to test the applicability of new FA treatment modalities, such as gene therapy [114-120], *ex vivo* manipulation of hematopoietic stem/progenitor cells [121] and cytokine treatment [122].

*Drosophila, Caenorhabditis elegans, Xenopus* and zebrafish homologs of FA genes have been more recently identified (Table 1). The FA gene network is conserved from zebrafish [123] and *Xenopus*[124] to human, while only *FANCD2, FANCL* and *FANCM* have *Drosophila* homologs [125], and *FANCD1/BRCA2, FANCD2 (FCD-2), FANCJ* and *FANCM* have homologs in *C. elegans*[126] (Table 1). *Drosophila* and *C. elegans* models will therefore be useful for testing the functions of key proteins in the FA pathway. As only FANCD2, FANCL and FANCM are present in *Drosophila*, these proteins may constitute the minimal FA pathway machinery. In turn, *C. elegans* may enable a better understanding of the roles of FANCD1/BRCA2 and FANCJ in the context of the minimal machinery constituted by FANCD2 and FANCM. As with vertebrate mutants, *Drosophila fancd2* and *fancl* mutants, as well as *C. elegans fancd2* mutants, show hypersensitivity to DNA crosslinking agents [125,126]. *Drosophila* and *C. elegans* models may, therefore, be useful to dissect the roles and regulations of the FA pathway in a less complex network. The zebrafish model may prove a valuable tool with which to investigate the impact of FA pathway failure on development as *fancd2*-deficient zebrafish embryos develop similar defects to those found in children with FA, including shortened body length, microcephaly (small head) and microphthalmia (small eyes) [127]. The *Xenopus* model mostly constitutes a powerful tool with which to investigate the regulation of the FA pathway *in vitro* through cell-free assay systems using replicating egg extracts [124]. Such assays could also be utilized for screening drugs that modulate the FA pathway.

## Disease targets and ligands

The FA pathway is required for cellular resistance to DNA crosslinking agents, including widely used anti-cancer drugs such as cisplatin, MMC and melphalan. As such, inhibition of the FA pathway will lead to sensitization of tumor cells to these drugs. Therefore, the FA pathway is an attractive target for developing small molecule inhibitors that may be clinically useful as chemosensitizers in the treatment of cancer. As described above, the FA pathway involves formation of a multi-subunit protein complex harboring E3 ligase activity, several enzymes (ubiquitin ligase and conjugating enzyme, deubiquitinating enzyme, kinase, ATPase/DNA translocase and ATPase/helicase) and many protein–protein or protein–DNA interactions. All of these components are potential targets for small molecule inhibitors of the FA pathway.

Drug development targeting the FA pathway is still in the early stages, and therefore not much information is available. A high-throughput cell-based screening assay for small molecule inhibitors of the FA pathway has, however, been developed by Alan D'Andrea (Dana-Farber Cancer Institute), Toshiyasu Taniguchi (Fred Hutchinson Cancer Research Center) and their colleagues [128]. In this screen, inhibition of DNA damage-induced FANCD2 nuclear foci formation was utilized as a readout. Continued screening is ongoing and a partial result focusing on one inhibitor, curcumin (diferuloylmethane), has been published [128]. Curcumin is a low-molecular-weight polyphenol derived from the rhizome *Curcuma longa* and is the principal ingredient in the spice turmeric [129]. Curcumin inhibits FANCD2 monoubiquitylation and nuclear foci formation, although its exact target in the FA pathway has not been identified [128]. However, curcumin sensitizes an ovarian cancer cell line to cisplatin in an FA pathway-dependent manner, suggesting that curcumin sensitization of cisplatin mostly occurs through FA pathway inhibition [128]. In order to establish curcumin as a useful cisplatin chemosensitizer, a detailed isobologram analysis of combinations of curcumin with cisplatin, *in vivo* studies using mouse models, and identification of the target of curcumin in the FA pathway are required.

## New frontiers in drug discovery

Elucidating the mechanism of action of the candidate inhibitors identified in the above-mentioned screen [128] will be crucial for development of specific and effective inhibitors of the FA pathway, and for further understanding the regulation of the FA pathway. Precise analyses of the effects of each drug on individual steps in the FA pathway will be required to identify their specific targets. These analyses will include *in vitro* ATR kinase assay, *in vitro* UBE2T and FANCL autoubiquitylation assays, assessment of FA core complex formation and evaluation of nuclear foci formation of DNA repair proteins including BRCA1. A better understanding of the regulatory mechanisms, as well as the identification of new partners of the FA pathway, is also crucial for the identification and development of FA pathway inhibitors. Although multiple groups have been working on structural analysis of FA proteins [130,131], so far few FA protein crystal structures (BRCA2/FANCD1 [130] and FANCF [131]) have been reported, but such studies could provide useful information for drug design as well as for elucidation of the targets of the candidate inhibitors.

## Abbreviations

FA: Fanconi anemia; MMC: mitomycin C; TLS: translesion synthesis; HR: homologous recombination; PCNA: proliferating cell nuclear antigen; PHD: plant homeodomain.

## Competing interests

The authors declare that they have no competing interests.

## Publication history

Republished from Current BioData's Targeted Proteins database (TPdb; ).
